# Integrating metabolomics and proteomics to identify novel drug targets for heart failure and atrial fibrillation

**DOI:** 10.1186/s13073-024-01395-4

**Published:** 2024-10-21

**Authors:** Marion van Vugt, Chris Finan, Sandesh Chopade, Rui Providencia, Connie R. Bezzina, Folkert W. Asselbergs, Jessica van Setten, A. Floriaan Schmidt

**Affiliations:** 1 Department of Cardiology, University Medical Center Utrecht, Utrecht University, Division Heart & Lungs, Utrecht, The Netherlands; 2 Institute of Cardiovascular Science, Faculty of Population Health, University College London, London, UK; 3 Department of Cardiology, Amsterdam Cardiovascular Sciences, Amsterdam University Medical Centre, University of Amsterdam, Amsterdam, Netherlands; 4 Amsterdam Cardiovascular Sciences, Heart Failure and Arrhythmias, Amsterdam, The Netherlands; 5 UCL British Heart Foundation Research Accelerator, London, UK; 6 Health Data Research UK and Institute of Health Informatics, University College London, London, UK; 7 Department of Experimental Cardiology, Amsterdam University Medical Centre, University of Amsterdam, Amsterdam, The Netherlands; 8 European Reference Network for rare, low prevalence and complex diseases of the heart: ERN GUARD-Heart , Amsterdam, The Netherlands; 9 Institute of Health Informatics, University College London, London, UK; 10 The National Institute for Health Research University College London Hospitals Biomedical Research Centre, University College London, London, UK

**Keywords:** Heart failure, Atrial fibrillation, Cardiomyopathy, Metabolomics, Drug development, Mendelian randomisation, Proteomics

## Abstract

**Background:**

Altered metabolism plays a role in the pathophysiology of cardiac diseases, such as atrial fibrillation (AF) and heart failure (HF). We aimed to identify novel plasma metabolites and proteins associating with cardiac disease.

**Methods:**

Mendelian randomisation (MR) was used to assess the association of 174 metabolites measured in up to 86,507 participants with AF, HF, dilated cardiomyopathy (DCM), and non-ischemic cardiomyopathy (NICM). Subsequently, we sourced data on 1567 plasma proteins and performed *cis* MR to identify proteins affecting the identified metabolites as well as the cardiac diseases. Proteins were prioritised on cardiac expression and druggability, and mapped to biological pathways.

**Results:**

We identified 35 metabolites associating with cardiac disease. AF was affected by seventeen metabolites, HF by nineteen, DCM by four, and NCIM by taurine. HF was particularly enriched for phosphatidylcholines (*p* = 0.029) and DCM for acylcarnitines (*p* = 0.001). Metabolite involvement with AF was more uniform, spanning for example phosphatidylcholines, amino acids, and acylcarnitines. We identified 38 druggable proteins expressed in cardiac tissue, with a directionally concordant effect on metabolites and cardiac disease. We recapitulated known associations, for example between the drug target of digoxin (AT1B2), taurine and NICM risk. Additionally, we identified numerous novel findings, such as higher RET values associating with phosphatidylcholines and decreasing AF and HF. RET is targeted by drugs such as regorafenib which has known cardiotoxic side-effects. Pathway analysis implicated involvement of GDF15 signalling through RET, and ghrelin regulation of energy homeostasis in cardiac pathogenesis.

**Conclusions:**

This study identified 35 plasma metabolites involved with cardiac diseases and linked these to 38 druggable proteins, providing actionable leads for drug development.

**Supplementary Information:**

The online version contains supplementary material available at 10.1186/s13073-024-01395-4.

## Background

Atrial fibrillation (AF) and heart failure (HF) are common types of cardiac disease, resulting in substantial mortality and morbidity, and placing a major burden on healthcare and society [1, 2]. Despite advances in management and understanding of disease pathophysiology, AF and HF prevalence remain high [1, 2]. HF is a particularly heterogeneous disease potentially caused by cardiomyopathies, such as dilated cardiomyopathy (DCM) and non-ischemic cardiomyopathy (NICM).

Currently, plasma measurements to inform cardiac disease management are limited to N-terminal prohormone of brain natriuretic peptide (NT-proBNP), troponin, and D-dimers. Specifically, cardiomyocytes release NT-proBNP into plasma upon wall stress and pressure overload and therefore NT-proBNP serves as an indirect biomarker of all-cause HF. NT-proBNP levels increase rapidly in acute HF and may remain elevated, reflecting continuous stress and correlating with disease severity [3]. D-dimers are produced during fibrin degradation following blood clot dissolution. High plasma levels suggest substantial clot formation indicative of pulmonary embolism [4]. Elevated plasma troponin levels result from myocardial damage and are primarily used in acute settings [5]. Diseased hearts and cardiomyocytes show altered metabolism, shifting to ketone oxidation and glycolysis [6–8]. Small, predominantly cross-sectional studies in AF and HF patients have reported elevated plasma values of acylcarnitines (ACs), which are markers of impaired fatty acid oxidation [9–15].

Metabolic changes characterising cardiac diseases can inform drug development, which is especially important for HF and AF, where currently available drugs do not necessarily address underlying aetiology. For example, no novel AF drugs have been approved since the 2011 introduction of the anticoagulants rivaroxaban, apixaban, and edoxaban, which are indicated for stroke prevention in AF patient without addressing the underlying cause of AF.

Both metabolites and proteins, the main targets of most drugs [16], are increasingly analysed by high-throughput assays measuring the plasma values of hundreds of analytes [17–19]. Genome-wide association studies (GWAS) have now identified genetic determinants of protein and metabolite values, which allow for integrative analyses identifying potential associations between the protein values, metabolite values, and cardiac disease in humans. Specifically, through two-sample Mendelian randomisation (MR), one can anticipate the effect an exposure (e.g. a metabolite) will have on the onset of disease by sourcing genetic variants strongly associated with the exposure and determining whether these variants show a dose–response association with disease [20, 21]. This approach has been extensively validated for cardiovascular disease [22–25], including a recent study on plasma proteins affecting cardiac function and structure as well as cardiac disease [26].

In the current study, we sourced data on 174 metabolites measured in up to 86,507 participants and performed genome-wide MR to identify metabolites associated with AF, HF, DCM, and NICM. Subsequently, we leveraged data on 1567 proteins measured in up to 35,559 participants and used *cis* MR to identify proteins affecting both the identified metabolites and the considered cardiac outcomes. We triangulated and prioritised proteins with concordant effects on metabolite values and cardiac outcomes. Through the integration of these omics data we identified 38 drugged and druggable proteins expressed in cardiac tissue, which affected 35 metabolites and cardiac disease.

## Methods

### Data sources

Genetic associations with 174 metabolites measured in up to 86,507 participants were available from a meta-analysis of Lotta et al. [27]. Genetic associations with AF were sourced from Nielsen et al. (60,620 cases) [28], with HF from Shah et al. (47,309 cases) [29], with DCM from Garnier et al. (2719 cases) [30], and with NICM from Aragam et al. (1816 cases) [31]. Genetic associations with plasma proteins were available from eight GWAS: deCODE (SomaLogic assay, *n* = 35,559) [32], SCALLOP (OLINK assay, *n* = 30,931) [33], Ahola-Olli et al*.* (BioRad assay, *n* = 8293) [34], Framingham (Luminex assay, *n* = 6861) [35], AGES-Reykjavik (SomaLogic assay, *n* = 5368) [36], INTERVAL (SomaLogic assay, *n* = 3301) [37], Gilly et al. (Olink assay, *n* = 1328) [38], and Yang et al. (SomaLogic assay, *n* = 636) [39]. Additional details are described in the Supplementary Note.

### Mendelian randomisation analyses

**Fig. 1:**
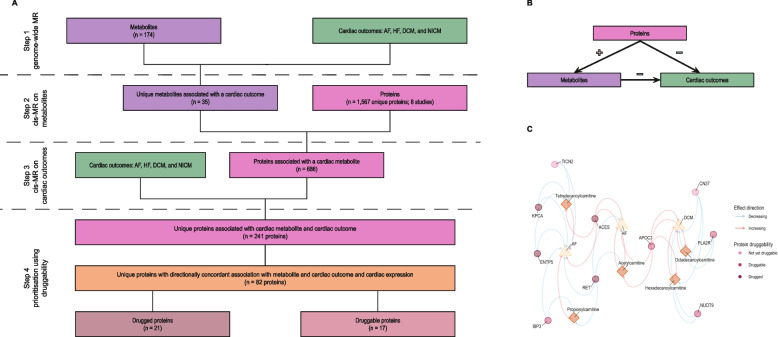
Study design to identify plasma proteins and metabolites affecting cardiac disease. **A** Flowchart of the analysis steps. **B** A triangulation diagram, illustrating how a robust set of directionally concordant proteins was identified affecting plasma metabolite levels as well as cardiac disease. The red plus indicates a risk-increasing effect, while the blue minus indicates a risk-decreasing effect. **C** Annotated network of prioritised metabolites, proteins, and outcomes for which the metabolites have at least 20% associated common proteins and belong to the metabolite class acylcarnitines. Prioritised directionally concordant proteins are represented by circles, metabolites by diamonds, outcomes by triangles. Circle colours represent protein druggability. Increasing effects are displayed by a red arrow, decreasing effects by a blue arrow. See Supplementary Note for more details. Abbreviations: AF = atrial fibrillation, DCM = dilated cardiomyopathy, HF = heart failure, MR = mendelian randomisation, NICM = non-ischemic cardiomyopathy

Genome-wide MR, considering genetic variants from across the genome, was used to establish associations between metabolite values and cardiac outcomes (step 1 in Fig. 1A, Table S1). We subsequently performed cis MR to identify the effects of plasma proteins on metabolite values and cardiac outcomes (steps 2–3 in Fig. 1A). When a protein was available in multiple studies, we a priori decided to use the largest sample size GWAS for the primary analysis, employing the smaller sample size data for replication of proteins with a significant association with cardiac disease. For the *cis* MR, we sourced genetic variants from a 200-kilobase pair window surrounding the protein-encoding gene [20]. Irrespective of the type of MR analysis, instruments were selected based on an exposure F-statistic of at least 24 and a minor allele frequency of at least 0.01. Additionally, variants were clumped to a linkage disequilibrium r-squared of 0.3, based on a random sample of 5000 unrelated UK biobank participants of European ancestry.

Analyses were conducted using the generalised least squares implementation of the inverse-variance weighted (IVW) estimator, as well as with an Egger correction protecting against horizontal pleiotropy [40]. Influence of horizontal pleiotropy was minimised by excluding variants with a leverage statistic larger than three times the mean, and by excluding variants with an outlier (i.e. chi-square) statistic larger than 10.83 [41]. To ensure we had sufficient data to accurately model the exposure effects, we discarded analyses with fewer than 6 variants. Additionally, a model selection strategy was used to select the most appropriate estimator (IVW or MR-Egger) [41, 42], where the MR-Egger is unbiased in the presence of directional horizontal pleiotropy. The model selection framework, originally developed by Rücker et al. [43], uses the difference in heterogeneity between the IVW Q-statistic and the Egger Q-statistic to decide which method provides the best model to describe the available data. Furthermore, due to the two-sample nature of our analyses, any potential weak-instrument bias would act toward a null effect, reducing power, rather than increasing type 1 errors [26].

### Effect estimates, multiple testing, and nomenclature

MR results are presented as mean difference (MD) for continuous outcomes, or odds ratios (ORs) for binary outcomes, accompanied by 95% confidence intervals (CIs) and *p*-values. Metabolite effects on disease were filtered for a multiplicity corrected *p*-value threshold of 7 × 10^−5^ based on the 174 available metabolites and four outcomes, which is conservative, considering many metabolites are correlated. Similarly, protein MR effect estimates were evaluated against a multiplicity corrected *p*-value of 9 × 10^−7^ for the metabolites and 2 × 10^−5^ for the cardiac outcomes, based on the number of tested proteins (1567 in the metabolite MR and 686 in the cardiac MR) and outcomes (36 in the metabolite MR and four in the cardiac MR). Proteins are referred to using their Uniprot label and presented in normal font to differentiate from gene names in italic font.

### Prioritisation and annotation of proteins

Proteins were prioritised based on the association with a plasma metabolite and a directionally concordant effect on cardiac outcome. For example, if an increase in a metabolite value decreased the risk of AF, a protein that increased the value of this metabolite should decrease AF risk to be deemed concordant. In this way, robust triangles of associations between the metabolites, proteins, and cardiac outcomes are created (Fig. 1B). Given that this triangulation considers evidence from three separate MR analyses with distinct underlying assumptions on horizontal pleiotropy, this approach identifies a robust set of results, highly supported by the available data. For example, *cis* MR analyses assume the absence of pre-translational pleiotropy, which may be more robust compared to more distal assumptions on horizontal pleiotropy employed in genome-wide MR [20].

The identified proteins were further annotated and prioritised on the presence of cardiac mRNA expression sourcing information from the human protein atlas (HPA) [44]. Subsequently, we identified genes which were overexpressed in cardiac tissue through comparisons against the average mRNA expression across the other 60 available tissues (Supplementary Note). We obtained information on druggability, indications and side-effects from ChEMBL v33 and the British National Formulary (BNF) identifying proteins as “druggable” if they are targeted by a developmental drug and “drugged” if they are targeted by an approved compound. Finally, the Reactome [45] knowledgebase was queried to map all 1567 proteins to biological pathways. Pathway enrichment was established by (1) calculating the proportion of prioritised proteins that were involved in the pathway, (2) calculating the total number of GWAS proteins involved in the pathway, (3) testing the difference between steps 1 and 2 against a standard normal distribution using a Wald-based procedure. Enrichment analysis was conducted using all prioritised proteins irrespective of the cardiac outcomes they were associated with in the MR analysis, in addition to disease-specific analyses.

### Replicating protein associations with metabolites and cardiac disease

The aforementioned MR analyses sourced data from the eight proteomic GWAS (deCODE, SCALLOP, Ahola-Olli, Framingham, AGES-Reykjavik, INTERVAL, Gilly, and Yang), selecting only the largest sample size GWAS for proteins present in multiple studies. In case a single protein was measured in multiple studies, we a priori decided to use the remaining protein GWAS to replicate the 49 prioritised proteins with data available in multiple studies. Replication was sought by identifying associations with the same effect direction as the discovery association, and a *p*-value smaller than 0.05 (nominal replication) and 0.05/49 = 0.001 (conservative replication).

## Results

### Metabolite effects on cardiac outcomes

**Fig. 2:**
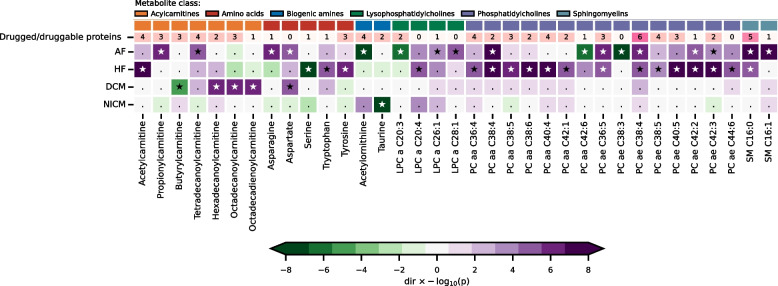
Plasma metabolites associating with at least one cardiac outcome. N.B. For visualisation purposes, the *p*-values were truncated to a −log(10) of 10. Multiplicity corrected significant associations are depicted by a star, non-significant associations by a dot. The second row indicates the number of druggable proteins reflecting information retrieved from BNF and ChEMBL. Genetic associations with the cardiac outcomes were obtained from Nielsen et al. (60,620 AF cases) [33], Shah et al. (47,309 HF cases) [34], Garnier et al. (2719 DCM cases) [35], and Aragam et al. (2038 NICM cases) [36]. See the “Methods” section additional details, and Appendix Table S2 for the underlying numerical data. Abbreviations: a = acyl residue, aa = diacyl residue, ae = acyl-alkyl residue, AF = atrial fibrillation, DCM = dilated cardiomyopathy, HF = heart failure, LPC = lysophosphatidylcholines, NICM = non-ischemic cardiomyopathy, PC = phosphatidylcholines, SM = sphingomyelins

Genome-wide MR identified 35 metabolites associating with one or more cardiac disease (Fig. 2, Table S2-3). Acylcarnitines (ACs) were enriched for DCM (*p* = 0.001; Table S4), with higher values of hexadecanoylcarnitine (OR 1.51, 95%CI 1.28; 1.79), octadecanoylcarnitine (OR 1.47, 95%CI 1.25; 1.73), and octadecadienoylcarnitine (OR 1.38, 95%CI 1.22; 1.55) associated with lower DCM risk, while higher values of butyrylcarnitine associated with higher DCM risk (OR 0.87, 95%CI 0.82; 0.93). Phosphatidylcholines (PCs) were enriched for HF (*p* = 0.029; Table S4) and all thirteen PCs associated with HF elicited a risk increasing effect. We observed more uniform effects for AF, which was affected by seventeen plasma metabolites. Finally, we observed that higher values of plasma taurine associated with lower NICM risk (OR 0.48, 95%CI 0.38; 0.60; Fig. 2).

### Protein effects on metabolite values and cardiac outcomes

**Fig. 3:**
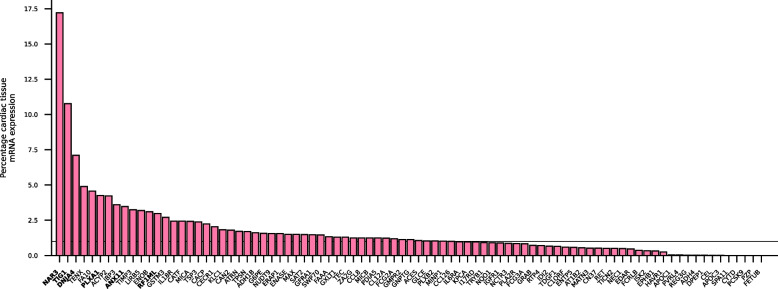
Percentage of mRNA expression in cardiac tissue of the subset of directionally concordant proteins affecting cardiac disease. N.B. The percentage mRNA expression in cardiac tissue was calculated by dividing cardiac expression by total expression in all organs. The horizontal line indicates 1% cardiac expression. Proteins are referred to using their Uniprot label and proteins in bold font are overexpressed in cardiac tissue relative to non-cardiac tissue. Data were sourced from the human protein atlas; see “Methods” section

We evaluated 1567 unique plasma proteins for associations with the 35 metabolites implicated in cardiac disease onset. This resulted in 686 proteins associated with one or more metabolite (step 2 in Fig. 1A, Fig S1). Prioritising proteins on an association with cardiac outcomes identified 241 proteins (step 3 in Fig. 1A, Fig S2), which were pruned down to 87 proteins by identifying the proteins with a directionally concordant effect on metabolites and cardiac outcomes (Table 1, Table S5-7). This triangulated subset of 87 proteins included 82 proteins that were expressed in cardiac tissue. Only CYTD, FETUB, PCSK9, PZP, and SPA11 were not expressed in cardiac tissue and six of the 82 prioritised proteins (ANX11, DNJA4, NAR3, PLXA1, RF1ML, and TIG1) were significantly overexpressed in the heart compared to other tissues (Fig. 3, Table S8).

**Table 1:** Overview and characteristics of drugged and druggable prioritised proteins and metabolites associated with atrial fibrillation (AF), heart failure (HF), dilated cardiomyopathy (DCM), or non-ischemic cardiomyopathy (NICM)

Uniprot ID	Protein	Druggability^a^	Metabolite class(es)^a^	No. metabolites^a^	Metabolite effect^a^	Outcome(s)	Cardiac effect^a^	No. drugs^a^	Cardiac indication or side-effect^a^
Q9UBX1	CATF	Drugged	AA	1	Increasing	HF	0.92 (0.90; 0.94)	2	-
P17655	CAN2	Drugged	AA, PC	2	Increasing	HF	1.21 (1.13; 1.29)	2	-
P22303	ACES	Drugged	AC	1	Increasing	AF	1.09 (1.05; 1.13)	23	Both
Q15661	TRYB1	Drugged	AC	1	Increasing	AF	1.02 (1.01; 1.03)	1	Indication
O75356	ENTP5	Drugged	AC	1	Increasing	AF	0.89 (0.86; 0.93)	1	-
P43155	CACP	Drugged	AC	1	Decreasing	DCM	3.15 (2.03; 4.90)	1	Indication
Q96F10	SAT2	Drugged	AC	3	Mixed	DCM	0.73 (0.64; 0.84)	1	Indication
P22303	ACES	Drugged	AC	1	Increasing	HF	1.06 (1.04; 1.08)	23	Both
P07949	RET	Drugged	AC, AA	2	Increasing	AF	0.94 (0.93; 0.95)	14	Side-effect
P17252	KPCA	Drugged	AC, PC	2	Increasing	AF	0.81 (0.74; 0.89)	5	Side-effect
P07949	RET	Drugged	AC, PC	5	Increasing	HF	0.97 (0.96; 0.98)	14	Side-effect
P16444	DPEP1	Drugged	BA	1	Decreasing	AF	0.97 (0.96; 0.98)	2	Both
P19835	CEL	Drugged	BA	1	Decreasing	AF	0.95 (0.93; 0.97)	1	-
P14415	AT1B2	Drugged	BA	1	Decreasing	NICM	1.33 (1.19; 1.49)	6	Both
Q96NY8	PVRL4	Drugged	BA	1	Decreasing	NICM	0.32 (0.20; 0.51)	1	-
Q8NFI3	ENASE	Drugged	LPC	1	Decreasing	AF	1.06 (1.03; 1.08)	12	Indication
P00742	FA10	Drugged	PC	2	Increasing	AF	1.11 (1.06; 1.15)	13	Both
P10144	GRAB	Drugged	PC	2	Increasing	HF	1.21 (1.11; 1.32)	0	-
P54762	EPHB1	Drugged	PC, AA	2	Mixed	HF	0.95 (0.93; 0.97)	1	-
P42680	TEC	Drugged	SM	1	Increasing	AF	0.87 (0.83; 0.90)	1	-
P08887	IL6RA	Drugged	SM	1	Increasing	AF	0.96 (0.96; 0.97)	7	Both
P08887	IL6RA	Drugged	SM	1	Increasing	HF	0.98 (0.97; 0.99)	7	Both
P15559	NQO1	Drugged	SM	1	Increasing	HF	0.97 (0.96; 0.98)	4	-
P21266	GSTM3	Drugged	SM, BA	2	Mixed	AF	1.04 (1.02; 1.05)	1	-
Q13018	PLA2R	Druggable	AA	2	Increasing	HF	1.01 (1.01; 1.02)	0	-
P17936	IBP3	Druggable	AC	1	Increasing	AF	0.93 (0.91; 0.96)	0	-
P02656	APOC3	Druggable	AC	2	Increasing	DCM	2.15 (1.62; 2.85)	0	-
Q13018	PLA2R	Druggable	AC	1	Increasing	DCM	0.92 (0.91; 0.94)	0	-
Q9BW91	NUDT9	Druggable	AC	1	Increasing	DCM	0.27 (0.15; 0.49)	0	-
P08637	FCG3A	Druggable	AC	1	Decreasing	DCM	1.07 (1.05; 1.10)	1	-
P00326	ADH1B	Druggable	AC	1	Increasing	HF	1.14 (1.08; 1.20)	2	-
Q13478	IL18R	Druggable	AC, LPC	2	Increasing	AF	0.95 (0.94; 0.97)	1	-
Q9H665	IGFR1	Druggable	BA	1	Decreasing	AF	1.05 (1.03; 1.08)	0	-
P61244	MAX	Druggable	LPC	1	Decreasing	AF	1.09 (1.05; 1.14)	0	-
Q9P2T1	GMPR2	Druggable	PC	2	Increasing	AF	0.93 (0.90; 0.96)	0	-
P56159	GFRA1	Druggable	PC	1	Increasing	AF	0.91 (0.89; 0.94)	1	-
Q9UNE0	EDAR	Druggable	PC	6	Increasing	HF	0.92 (0.88; 0.95)	0	-
P12318	FCG2A	Druggable	PC	1	Increasing	HF	0.99 (0.98; 0.99)	0	-
Q9NZ08	ERAP1	Druggable	PC	2	Increasing	HF	0.97 (0.96; 0.98)	1	-
P29120	NEC1	Druggable	PC	3	Increasing	HF	0.97 (0.96; 0.99)	0	-
P08319	ADH4	Druggable	PC	5	Increasing	HF	0.70 (0.61; 0.80)	1	-
P08637	FCG3A	Druggable	PC	1	Increasing	HF	1.01 (1.01; 1.02)	1	-
P02656	APOC3	Druggable	PC, AC	5	Increasing	HF	1.29 (1.18; 1.41)	0	-
P29120	NEC1	Druggable	PC, SM	3	Increasing	AF	0.96 (0.94; 0.97)	0	-
P13385	TDGF1	Druggable	SM, PC	2	Mixed	AF	1.03 (1.02; 1.04)	1	-

^a^Columns: Druggability—whether the protein is targeted by a developmental (“druggable”) or approved (“drugged”) compound, Metabolite class(es)—metabolite class(es) of the metabolites with which the indicated protein is associated, No. metabolites—number of metabolites the protein is associated with, Metabolite effect—effect of protein on metabolites, defined as increasing if higher values of the protein increase the plasma metabolite values, decreasing if higher values of the protein decrease the metabolite values, and mixed if higher values of the protein increase values of some and decrease values of other metabolites, Cardiac effect—odds ratio (95% confidence interval) of the association between the protein and atrial fibrillation (AF), No. drugs—number of drugs that target the indicated protein, Cardiac drug effect—whether at least one drug has a cardiac indication, side-effect, or both as indicated by BNF or ChEMBL

*Abbreviations*: *AA* Amino acids, *AC* Acylcarnitines, *AF* Atrial fibrillation, *BA* Biogenic amines, *DCM* Dilated cardiomyopathy, *HF* Heart failure, *LPC* Lysophosphatidylcholines, *NICM* Non-ischemic cardiomyopathy, *PC* Phosphatidylcholines, *SM* Sphingomyelins

**Fig. 4:**
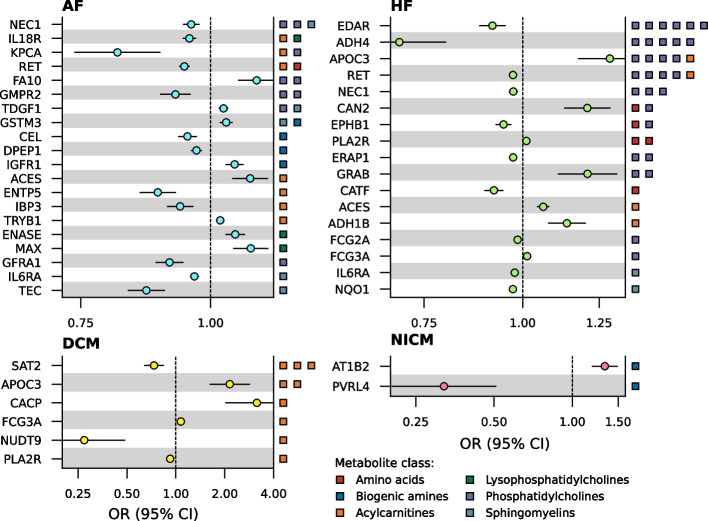
Forest plots of prioritised drugged or druggable proteins effect on cardiac outcome. N.B. Metabolite class of the metabolites affected by the protein are indicated on the right *y*-axis. Genetic associations with the cardiac outcomes were obtained from Nielsen et al. (60,620 AF cases) [33], Shah et al. (47,309 HF cases) [34], Garnier et al. (2719 DCM cases) [35], and Aragam et al. (2038 NICM cases) [36]. See the “Methods” section for a more detailed description and Appendix Table S7 for the full numerical results. Abbreviations: AF = atrial fibrillation, CI = confidence interval, DCM = dilated cardiomyopathy, HF = heart failure, NICM = non-ischemic cardiomyopathy, OR = odds ratio

ChEMBL and BNF were consulted to identify seventeen proteins that were druggable (i.e. targeted by a developmental compound) and 21 that were drugged (i.e. targeted by an approved compound; Fig. 4, Table S9-10). Eleven of the 21 drugged proteins were targeted by drugs with a cardiac indication, side-effect, or both. For example, higher values of AT1B2 (targeted by HF drug digoxin) associated with higher NICM risk and decreased plasma taurine values. Furthermore, higher values of DPEP1 (associated with acetylornithine values) and KPCA (associated with tetradecanoylcarnitine and PC ae C38:4 values) associated with lower AF risk, while higher values of FA10 (associated with PC ae C42:2 and PC ae C38:4 values) associated with higher AF risk (Fig. 4). DPEP1 is inhibited by cilastatin, which is indicated for endocarditis and is implicated with tachycardia, KPCA is inhibited by midostaurin, which is known to elicit QT-interval prolongation, and FA10 is inhibited by drugs like apixaban, edoxaban and rivaroxaban, which are commonly used to prevent stroke in AF. The remaining ten drugged proteins were targeted by compounds indicated for treatment of cancers (CAN2, EPHB1, ERAP1, GSTM3, NQO1, and PVRL4), mitochondrial and muscular disease (NQO1), and metabolic syndrome (CEL; Table S10).

Irrespective of the indication or reported side-effect, drugged or druggable proteins associating with HF frequently affected phosphatidylcholines values. Higher values of ADH4 (targeted by nitrefazole), EDAR, EPHB1 (targeted by cancer drug vandetanib), ERAP1 (targeted by cancer drug tosedostat), and FCG2A associated with lower HF risk, while higher values of APOC3, CAN2 (targeted by amyloidosis drugs bortezomib and carfilzomib), and GRAB associated with higher HF risk (Fig. 4, Table S7). DCM was affected by six drugged or druggable proteins, all affecting one or more acylcarnitines. Higher values of SAT2 (targeted by trientine, which is in phase 2 clinical development for hypertrophic cardiomyopathy) and NUDT9 associated with lower DCM risk, while FCG3A (the target of imgatuzumab) and CACP (targeted by levocarnitine, which is being tested for AF and HF) associated with higher DCM risk. Higher values of CEL (targeted by obesity medicine orlistat), ENTP5 (targeted by the psoriasis drug anthralin), GMPR2, GFRA1 (targeted by liatermin, a phase 1 compound for Parkinson’s disease), IBP3 and TEC (targeted by Pf-06651600, which is in clinical testing for arthritis, kidney and Crohn’s disease) associated with lower AF risk, while ENASE (targeted by AF drug edoxaban), GSTM3 (targeted by carmustine), IGFR1, TDGF1 (targeted by the antibody biib-015 used to treat cancer), and TRYB1 (targeted by arginine, which is being tested for HF) associated with higher AF risk, affecting metabolites of various classes (Fig. 4, Table 1, Table S7). Four drugs target more than one prioritised protein: Cep-2563 targets KPCA and RET, edoxaban targets ENASE and FA10, nitrefazole targets ADH1B and ADH4, and vandetanib targets EPHB1 and RET (Table S9).

**Fig. 5:**
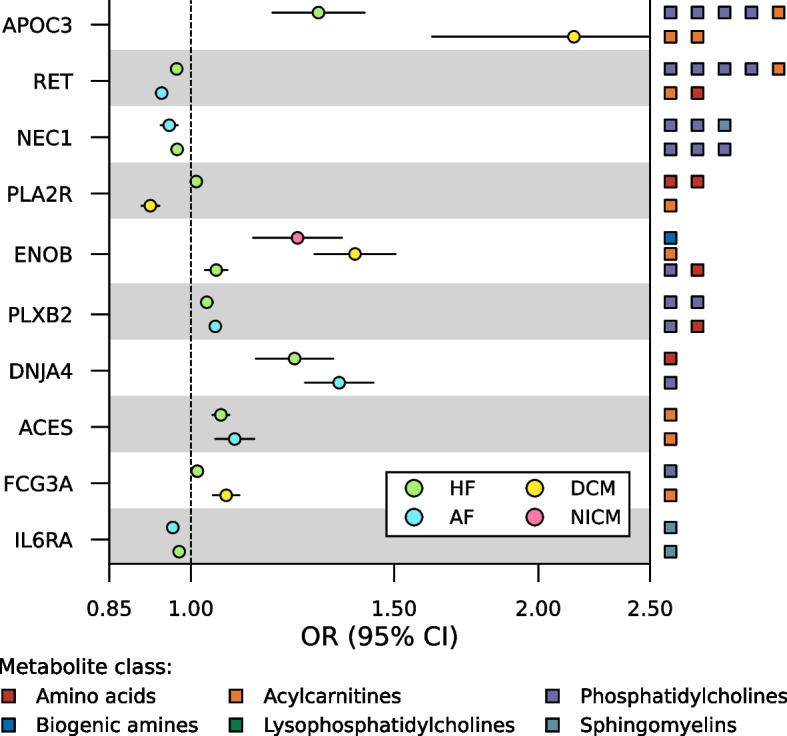
Forest plot of prioritised proteins associating with more than one cardiac outcome. N.B. Metabolite class of the metabolites affected by the protein are indicated on the right *y*-axis. Genetic associations with the cardiac outcomes were obtained from Nielsen et al. (60,620 AF cases) [33], Shah et al. (47,309 HF cases) [34], Garnier et al. (2719 DCM cases) [35], and Aragam et al.(2038 NICM cases) [36]. See the “Methods” section for a more detailed description and Appendix Table S7 for the full numerical results. Abbreviations: AF = atrial fibrillation, CI = confidence interval, DCM = dilated cardiomyopathy, HF = heart failure, NICM = non-ischemic cardiomyopathy, OR = odds ratio

Ten pleiotropic proteins affected multiple cardiac outcomes (Fig. 5). For example, higher values of ACES (inhibited by pyridostigmine bromide, which is in phase 2 testing for HF), DNJA4 and PLXB2 associated with higher AF and HF risk, whereas higher values of IL6RA (targeted by tocilizumab), NEC1, and RET (targeted by regorafenib, which has cardiac side-effects) associated with lower AF and HF risk. Higher values of APOC3 and FCG3A associated with higher HF and DCM risk. ENOB associated with higher risk of HF (OR 1.05, 95%CI 1.03; 1.08), DCM (OR 1.39, 95%CI 1.28; 1.50), and NICM (OR 1.24, 95%CI 1.13; 1.35; Fig. 5, Table S7).

### Identifying enriched biological pathways

We used the Reactome [45] knowledgebase to identify enriched pathways amongst the prioritised proteins which identified three enriched pathways involving seven of the prioritised proteins. Specifically, GFRA1, KPCA, and RET were involved in the “RET signalling” pathway, TPSN and ERAP1 in “antigen presentation: folding, assembly, and peptide loading of class I MHC”, and ACES and NEC1 in the “synthesis, secretion, and deacylation of ghrelin” pathway (Table S11). Reactome pathway enrichment results per cardiac outcome are depicted in Fig S3.

### Replicating protein associations with cardiac disease

Replication data from independent plasma protein data were available for 49 out of 82 prioritised proteins. Applying a nominal replication *p*-value of 0.05, we were able to replicate the cardiac association of 38 proteins (77.6%; Table S12). The effects of 24 proteins were replicated more than once, with the IL6RA effects on AF and HF replicated in up to five studies. Applying a multiple testing adjusted *p*-value cut-off, resulted in 30 replicated proteins (61.2%), also including IL6RA. Please see the Supplementary Note for replication of the protein association with plasma metabolite values.

## Discussion

In the current study, we integrated genetic associations with 174 metabolites and 1567 proteins to identify determinants of cardiac disease relevant for de novo drug development and drug repurposing. Results were prioritised on concordant effect direction and cardiac tissue expression, identifying 82 triangulated proteins and 35 metabolites associating with AF, HF, DCM, and NICM. Plasma acylcarnitine (AC) values were strongly related with DCM, while phosphatidylcholine (PC) values were important determinants of HF. Metabolites affecting AF spanned six metabolite classes, potentially reflecting the heterogeneous aetiology of AF. We identified ten pleiotropic proteins affecting multiple cardiac traits: ACES, APOC3, DNJA4, ENOB, FCG3A, IL6RA, NEC1, PLA2R, PLXB2, and RET. The 38 drugged and druggable proteins included eleven proteins with a known cardiac indication, side-effect, or both: ACES (donepezil), AT1B2 (digoxin), CACP (levocarnitine), DPEP1 (cilastatin), ENASE (edoxaban), FA10 (apixaban), IL6RA (tocilizumab), KPCA (midostaurin), RET (regorafenib), SAT2 (trientine), and TRYB1 (arginine). Pathway enrichment identified proteins involved in GDF15-dependent RET signalling, ghrelin homeostasis, and MHC I antigen presentation.

ACs were strongly associated with DCM onset, with prioritised proteins affecting DCM also associating with long-chain (hexadecanoylcarnitine, octadecanoylcarnitine, octadecadienoylcarnitine) and short-chain (butyrylcarnitine) AC values (Table 1, Table S7). Higher AC values were associated with a higher AF risk (propionylcarnitine and tetradecanoylcarnitine), and HF (acetylcarnitine). AC values are indicators of fatty acid oxidation, the main energy substrate of the healthy heart [8], with particularly long-chain ACs the results of fatty acid metabolism, while short-chain ACs are more generally synthesised during glucose, AA and fatty acid metabolism [46]. Fourteen drugged and druggable proteins associated with AC plasma values (Table S7). We showed that ACES affects the plasma values of several ACs and was associated with a risk increasing effect on AF and HF. Pyridostigmine bromide is an ACES inhibitor, which is being tested for HF treatment (Table S9) and it increases heart rate variability and reduces ventricular arrhythmias in chronic HF patients [47]. These findings therefore support the identified associations with HF, but also AF, for which heart rate variability is a predictor [48, 49]. ACES is also targeted by other drugs with cardiac side effects, making its inhibition important to manage. NEC1 affects several PCs, AF, and HF, and is a druggable protein that processes hormones such as insulin and its encoding gene *PCSK1* associated with HF risk factors such as obesity [50] and abnormal glucose homeostasis [51]. NEC1 and ACES are involved in the enriched ghrelin pathway (Table S11), which regulates energy homeostasis and plays a role in myocardial function [52–55]. Ghrelin was therefore proposed as a novel target for HF management [56].

We identified thirteen PCs that increased HF risk, of which five also associated with higher AF risk. These results suggest that PCs play an important role in cardiac function, which is supported by the findings that PCs are implicated in aberrant calcium handling [57, 58]. We identified sixteen drugged and druggable proteins affecting PC values as well as AF or HF, or both (Table S7). Higher ERAP1 values (targeted by tosedostat) were shown to be associated with lower plasma PC values and HF risk, is targeted by cancer drug tosedostat, and plays a role in blood pressure regulation [59]. Together with the not yet druggable protein TPSN, ERAP1 is involved in the enriched MHC I antigen presentation pathway which is linked to CD8 + T-cell-mediated cardiac remodelling leading to HF [60]. We additionally found that higher KPCA values associated with lower AC and PC values and lower risk of AF. KPCA is targeted by HF drug midostaurin and is involved in the significantly enriched RET signalling pathway, together with GFRA1 and RET (Table S11). The RET receptor is activated by GFRA1 and is needed for GDF15 signalling [61], where GDF15 concentrations are predictive of HF outcome [62, 63]. GDF15 has also been shown to associate with stroke, bleeding, and mortality in AF patients [64]. RET-inhibiting cancer drug regorafenib was found to induce cardiac ischemia [65], which would create a substrate for arrhythmia, thus increasing AF and HF risk. Our observation that increased RET values are protective against AF and HF may indicate these adverse effects are on-target (Table S9, Figs. 4 and 5).

Except for PLA2R, all proteins associated with more than one cardiac outcome showed consistent effect direction across the outcomes (Fig. 5), emphasising their potential as drug targets. PLA2R interacts with both collagen-I and the pro-inflammatory enzyme phospholipase A2, leading to different effects depending on whether PLA2R is soluble or membrane-bound. The soluble form of PLA2R has been shown to reduce fibrosis by binding to collagen-I and inhibiting its interaction with integrin β1. This beneficial effect is counterbalanced by impairing the healing process of infarcted regions post-myocardial infarction. In contrast, the binding of collagen-I to membrane-bound PLA2R promotes collagen-I interaction with integrin β1 and thereby increases fibrosis [66, 67]. These opposing effects of PLA2R may help explain its seemingly contradictory effects on HF (risk-increasing) and DCM (risk-decreasing). These complex effects of the different forms of PLA2R on the heart suggest that further investigation is needed to fully understand its mechanism and to explore potential therapeutic approaches. However, one could speculate that these opposing effects could possibly be managed by specifically targeting the correct form or prescribing drug combinations.

Several approaches using publicly available datasets have been applied to identify de novo drug targets and repurposing opportunities. For example, our group recently employed deep-learning to derive sixteen cardiac MRI traits and used GWAS and plasma proteomics to identify 33 proteins associating with these MRI traits and cardiac outcomes [26]. In contrast, Kukendrarajah et al. conducted a systematic review combining MEDLINE with Open Targets to prioritise genetic loci associating with druggable targets for AF [68]. Henry et al. prioritised seven plasma proteins for HF by identifying proteins with an observational association between protein level and future HF risk, followed by confirmatory analysis using *cis* MR [69]. Our study uniquely adds to these complementary efforts by (1) performing a triangulation analysis to identify a robust subset of protein and metabolomic pathways relevant to cardiac disease, (2) providing evidence of tissue involvement, and (3) identifying existing evidence for cardiac indications or side-effects relevant for potential drug repurposing or indication expansion. As such, our analyses provide guidance for future wet-lab validation of targets such as RET and PLA2R, which will provide additional insights in whether the identified associations pertain to curative (i.e., reversable) or preventative (i.e., irreversible) disease mechanism.

We wish to discuss the following potential limitations. Firstly, our analyses focussed on plasma metabolites and proteins, which may not necessarily reflect in cardiac tissue values [70]. Evidence suggests that plasma values may serve as biomarkers of intracellular energy metabolism [12, 71] and correlate with metabolite values in cardiac tissues, for example cardiac profiles of medium- and long-chain ACs have been found to reflect plasma concentrations [46]. This is further supported by our observation that 82 of the 87 triangulated plasma proteins were expressed in cardiac tissue. Some of the proteins had minimal cardiac expression, amongst which also positive control AT1B2, which is targeted by HF-drug digoxin. While the current analysis prioritised findings on cardiac expression, proteins with expression in other organs may nevertheless exert an influence on cardiac disease. PCSK9 for example, which is targeted by approved monoclonal antibodies indicated for coronary heart disease, is predominantly expressed in the liver. Nevertheless, inhibition of PCSK9 reduces plasma concentration of low-density lipoprotein cholesterol, atherosclerosis and coronary heart disease [24, 72–75], which likely explains its association with heart failure. A second potential limitation is our reliance on high-throughput assays, which differ in terms of accuracy and coverage [76] and measure relative protein or metabolite values rather than concentrations. This means that the reported effect estimates and their magnitudes may not directly translate to anticipated clinical effects, hence small effects may nevertheless mark potentially clinically impactful targets. Additionally, while MR is a powerful tool to establish effect direction, providing information on the type of perturbation (activating versus inhibiting), it does not provide detailed understanding of the biological mechanism through which these exposures affect outcomes. Instead, our study provides a robust and prioritised list of targets for follow-up in wet-lab experiments. These experiments can elucidate disease pathophysiology and determine whether the identified associations are due to reversible or irreversible mechanisms. This distinction is crucial for determining whether a target is relevant for treatment of patients (in the case of reversible mechanisms) or better suited for prevention in people at elevated risk of future disease (in the case of irreversible mechanisms). Importantly, by establishing a list of highly viable targets, our work aims to enhance the efficiency and yield of subsequent wet-lab experiments. Third, while MR studies are susceptible to bias through horizontal pleiotropy, we mitigate this source of bias by removing potentially pleiotropic variants, employing a model selection framework, and triangulating results across analysis with distinct pleiotropy assumptions. Specifically, our triangulation approach minimised the potential for horizontal pleiotropy, by looking for agreement across three distinct MR analyses which also made distinct assumptions on horizontal pleiotropy. The robustness of this approach is highlighted by the rediscovery of multiple cardiac drug targets such as edoxaban, apixaban and rivaroxaban for AF and digoxin for HF. The triangulation of AT1B2 with taurine and NICM is particularly illustrative, because taurine supplementation may be used to replace or reduce the dosage of digoxin prescription in HF [77]. Finally, we emphasise that we were able to replicate associations for up to 77.6% of the proteins available in multiple studies.

## Conclusions

In conclusion, we identify 35 metabolites and 38 drugged or druggable proteins associating with AF, HF, DCM, or NICM. We were able to relate these findings to biological pathways relating to energy homeostasis through ghrelin and GDF15 signalling. Our findings provide evidence for relevant metabolites and proteins in cardiac diseases, pointing to involved pathways and providing relevant target prioritisation for pre-clinical drug development.

## Supplementary Information

Supplementary Material 1.

Supplementary Material 2. Table S1. Details of the metabolites considered. Table S2. Full MR results testing for the effect of metabolites on cardiac outcomes. Table S3. Number of metabolites per class associated with cardiac outcomes. Table S4. Enrichment of significant metabolite classes per cardiac outcome. Table S5. Number of overlapping prioritised proteins per outcome. Table S6. Overview and characteristics of not yet druggable prioritised proteins and metabolites associated with atrial fibrillation, heart failure, dilated cardiomyopathy, or non-ischemic cardiomyopathy. Table S7. Final results of all MRs per prioritised protein. Table S8. HPA RNA over-expression in heart. Table S9. Druggability results of the prioritised proteins. Table S10. Frequency of indication and side-effects. Table S11. Reactome pathway enrichment. Table S12. Replicates of the proteins associated with cardiac outcomes. Table S13. Replicates of the proteins associated with metabolites.

## Data Availability

All scripts and aggregated data are available online at https://gitlab.com/mvvugt/mr_metabolites_pub. The GWAS data on plasma metabolite values can be accessed via https://www.ebi.ac.uk/gwas/publications/33414548(27). The individual GWAS data on cardiac outcomes leveraged in this study can be accessed as follows: atrial fibrillation (cases = 60,620, total n = 1,030,836; https://www.ebi.ac.uk/gwas/publications/30061737(28)), heart failure (cases = 47,309, total n = 977,323; https://www.ebi.ac.uk/gwas/publications/31919418(29)), dilated cardiomyopathy (cases = 2,719, total n = 6,980; https://www.ebi.ac.uk/gwas/publications/33677556(30)) and non-ischemic cardiomyopathy (cases = 1,816, total n = 395,972; https://www.ebi.ac.uk/gwas/publications/30586722(31)). The individual GWAS data on plasma protein values can be accessed as follows: deCODE (n = 35,559, https://www.decode.com/summarydata/(32)), SCALLOP (n = 30,931, https://www.ebi.ac.uk/gwas/publications/33067605(33)), Ahola-Olli et al. (n = 8,293, https://www.ebi.ac.uk/gwas/publications/27989323(34)), Framingham (n = 6,861, https://www.ebi.ac.uk/gwas/publications/30111768(35)), AGES-Reykjavik (n = 5,368, https://www.ebi.ac.uk/gwas/publications/35078996(36)), INTERVAL (n = 3,301, https://www.ebi.ac.uk/gwas/publications/29875488(37)), Gilly et al. (n = 1,328, https://www.ebi.ac.uk/gwas/publications/33303764(38)), and Yang et al. (n = 636, https://www.ebi.ac.uk/gwas/publications/34239129(39)). Information on the variant-specific data per exposure-outcome pair for the considered Mendelian randomisation steps are available online at https://gitlab.com/mvvugt/mr_metabolites_pub.

## Abbreviations

AA: Amino acids
AC: Acylcarnitines
AF: Atrial fibrillation
BNF: British National Formulary
CI: Confidence interval
DCM: Dilated cardiomyopathy
EGFR: Epidermal growth factor receptor
GWAS: Genome-wide association studies
HF: Heart failure
HPA: Human protein atlas
IQR: Interquartile range
IVW: Inverse-variance weighted
LPC: Lysophosphatidylcholines
MD: Mean difference
MR: Mendelian randomisation
NICM: Non-ischemic cardiomyopathy
nTPM: Normalised transcripts per million
NT-proBNP: N-terminal prohormone of brain natriuretic peptide
OR: Odds ratio
PC: Phosphatidylcholines
SM: Sphingomyelins
