# Supplementary material for: Integrating metabolomics and proteomics to identify novel drug targets for heart failure and atrial fibrillation
Source: Genome Med. 2024 Oct 21;16:120. doi: 10.1186/s13073-024-01395-4 (PMC11492627; doi:10.1186/s13073-024-01395-4)
Supplement: Supplementary file 1 — Supplementary Material 1. [file 13073_2024_1395_MOESM1_ESM.pdf]

Appendix

Content

Supplementary Note……………………...………………………………………………………2
References……………………………………………………………………………………6
Figure legends……………………...…………………………………………………………7
Fig S1…………………………...……………………………………………………………10
Fig S2………………………………...………………………………………………………11
Fig S3………………………………..……………………………………………………….12
Fig S4……………………………..………………………………………………………….13
Fig S5…………………………………………………………………………………………14
Fig S6……………………………..………………………………………………………….15
Fig S7……………………………..……………………………………………………..….16
Fig S8……………………………..……………………………………………………..….17

Supplementary Note

Methods

Data sources

Genetic associations with the metabolites were sourced from Lotta *et al*. and the metabolites are grouped in the following classes: amino acids (AAs), biogenic amines (Bas), acylcarnitines (ACs), phosphatidylcholines (PCs), lysophosphatidylcholines (LPCs), sphingomyelins (SMs), and hexoses.

Genetic associations (i.e., point estimates and standard errors) with clinical outcomes were obtained from genome-wide association studies on atrial fibrillation (AF), heart failure (HF), dilated cardiomyopathy (DCM), and non-ischemic cardiomyopathy (NICM). Below, we summarize the study-specific characteristics.

- AF from Nielsen *et al.*(1), enrolling 1,030,836 European participants, including 60,620 cases. This meta-analysis included five prospective studies with study-specific definitions of AF:
    - ICD-10/9 codes I48 and 427.3 for the HUNT study.
    - ICD-9 code 427.31 for the Michigan Genomics Initiative.
    - ICD-10 code I48 for the DiscovEHR Collaboration Cohort.
    - ICD-10/9 codes I48 and 427.3 for the UK biobank.
    - A combination of EHR diagnosis (e.g., using ICD codes) and ECG/Holter measurements for the Christophersen *et al*. 2017 GWAS.
- HF from Shah *et al.*(2), enrolling 977,323 participants, including 47,309 cases. This meta-analysis of 26 studies defined HF a clinical diagnosis of HF of any aetiology with no inclusion criteria based on LV ejection fraction. The studies involved both case-control studies as well as population-based studies.

- DCM from Garnier *et al*.(3), enrolling 7,159 participants, including 2,719 cases. This meta-analysis of nine studies excluded the UK biobank and defined DCM based on the presence of reduced ejection fraction and an enlarged left ventricular end-diastolic volume/diameter in the absence of significant coronary artery disease, intrinsic valvular disease, documented myocarditis, systemic disease (such as sarcoidosis), sustained arterial hypertension, or congenital malformation.
- NICM from Aragam *et al*.(4), enrolling 395,972 participants, of whom 1,816 cases. This GWAS was exclusively conducted in the UK biobank, with NICM cases defined as an HF diagnosis with LV dysfunction in the absence of CAD. LV dysfunction was defined as a diagnosis of DCM or LV failure. CAD was defined as a diagnosis of myocardial infarction or having received a coronary revascularization.

Genetic associations (i.e., point estimates and standard errors) with protein levels were obtained from eight genome-wide association studies, which are summarized here:

- deCODE(5) measured plasma protein levels in 35,559 participants using a SomaLogic assay of 4,907 aptamers. The participants were mainly obtained by the Icelandic Cancer Project and the population-based deCODE Health study.
- The meta-analysis of SCALLOP(6) in 21,758 participants from 13 cohorts of European ancestry and 9,173 participants from two replication cohorts comprised the Olink CVD-I proteins.
- Ahola-Olli *et al*.(7) included 8,293 participants from three independent population-based Finnish studies. *et al*. and measured cytokine levels using BioRad assays.
- Framingham(8) measured 85 plasma protein levels using a Luminex assay in 6,861 participants of the prospective community-based FHS Offspring and Third Generation cohorts.
- The AGES-Reykjavik(9) study consisted of a random population of 5,368 participants living in Reykjavik in 1967 and their plasma protein levels were measured using 4,782 aptamers of the SomaLogic assay.

- The INTERVAL(10) substudy consisted of 3,301 blood donors from England and measured plasma protein levels using 4,034 aptamers of the SomaLogic assay.
- Gilly *et al.*(11) measured plasma protein levels of the CVDII, CVDIII and Metabolism Olink assays in 1,328 participants.
- Yang *et al.*(12) included 636 participants and measured plasma protein levels using 1,305 aptemers of the SomaLogic assay.

mRNA expression and enrichment

Cardiac mRNA expression was obtained from the human protein atlas (HPA), sourcing the consensus expression obtained by normalised transcripts per million (nTPM) values from three independent transcriptomics datasets: GTEx, Fantom5, and HPA's own data. Overexpressed genes were identified by comparing cardiac expression with average expression in other tissues, testing against a standard normal quantile of 1.96. Enrichment analysis, evaluating the frequency of association across metabolite classes was conducted using Fisher's exact.

Networks

We identified metabolites clusters based on a shared protein profile (defined as 20% or more protein in common) and visualised the triangulated associations using annotated networks. Reactome pathway enrichment of the proteins belonging to the metabolite networks compared to the remaining set of proteins was tested using a Wald test.

Results

Replicating protein associations with metabolites

Out of the 82 prioritised proteins, 49 were available in more than one GWAS, allowing for replication of the associations with plasma metabolites. Applying a nominal replication p-value of 0.05 we were able to replicate the metabolite association of 45 proteins (91.8%; **Table S13**). Applying a more stringent p-value cut-off resulted in 38 replicated proteins.

Networks

Cluster analysis was performed to identify plasma metabolites sharing common protein effects, identifying four groups (**Figure 1C**, **Fig S4-7**). This highlighted the following biological pathways: “TP53 regulates transcription of cell death genes” and “metabolism of RNA” (**Fig S8**).

References

1. Nielsen JB, Thorolfsdottir RB, Fritsche LG, Zhou W, Skov MW, Graham SE, et al. Biobank-driven genomic discovery yields new insight into atrial fibrillation biology. Nat Genet. 2018;50(9):1234–9.

2. Shah S, Henry A, Roselli C, Lin H, Sveinbjörnsson G, Fatemifar G, et al. Genome-wide association and Mendelian randomisation analysis provide insights into the pathogenesis of heart failure. Nat Commun. 2020;11(1):1–12.

3. Garnier S, Harakalova M, Weiss S, Mokry M, Regitz-Zagrosek V, Hengstenberg C, et al. Genome-wide association analysis in dilated cardiomyopathy reveals two new players in systolic heart failure on chromosomes 3p25. 1 and 22q11. 23. Eur Heart J. 2021;42(20):2000–11.

4. Aragam KG, Chaffin M, Levinson RT, McDermott G, Choi SH, Shoemaker MB, et al. Phenotypic refinement of heart failure in a national biobank facilitates genetic discovery. Circulation. 2019;139(4):489–501.

5. Ferkingstad E, Sulem P, Atlason BA, Sveinbjornsson G, Magnusson MI, Styrmisdottir EL, et al. Large-scale integration of the plasma proteome with genetics and disease. Nat Genet. 2021;53(12):1712–21.

6. Folkersen L, Gustafsson S, Wang Q, Hansen DH, Hedman ÅK, Schork A, et al. Genomic and drug target evaluation of 90 cardiovascular proteins in 30,931 individuals. Nat Metab. 2020;2(10):1135–48.

7. Ahola-Olli AV, Würtz P, Havulinna AS, Aalto K, Pitkänen N, Lehtimäki T, et al. Genome-wide association study identifies 27 loci influencing concentrations of circulating cytokines and growth factors. Am J Hum Genet. 2017;100(1):40–50.

8. Yao C, Chen G, Song C, Keefe J, Mendelson M, Huan T, et al. Genome-wide mapping of plasma protein QTLs identifies putatively causal genes and pathways for cardiovascular disease. Nat Commun. 2018;9(1):3268.

9. Gudjonsson A, Gudmundsdottir V, Axelsson GT, Gudmundsson EF, Jonsson BG, Launer LJ, et al. A genome-wide association study of serum proteins reveals shared loci with common diseases. Nat Commun. 2022;13(1):480.

10. Sun BB, Maranville JC, Peters JE, Stacey D, Staley JR, Blackshaw J, et al. Genomic atlas of the human plasma proteome. Nature. 2018;558(7708):73–9.

11. Gilly A, Park YC, Png G, Barysenka A, Fischer I, Bjørnland T, et al. Whole-genome sequencing analysis of the cardiometabolic proteome. Nat Commun. 2020;11(1):6336.

12. Yang C, Farias FH, Ibanez L, Suhy A, Sadler B, Fernandez MV, et al. Genomic atlas of the proteome from brain, CSF and plasma prioritizes proteins implicated in neurological disorders. Nat Neurosci. 2021;24(9):1302–12.

Figure legends

Fig S1. Volcano plots displaying proteins associated with the metabolites.

NB. Labelled proteins are drugged, which is defined as proteins targeted by a compound (see Methods); the p-value was truncated to a -log10 of 16 for visualisation purposes only. Abbreviations: a = acyl residue, aa = diacyl residue, ae = acyl-alkyl residue, LPC = lysophosphatidylcholine, MD = mean difference, PC = phosphatidylcholine, SM = sphingomyelin.

Fig S2. Volcano plots displaying proteins associated with the cardiac outcomes.

NB. Labelled proteins are drugged, which is defined as proteins targeted by a compound (see Methods); the p-value was truncated to a -log10 of 16 for visualisation purposes only. Abbreviations: AF = atrial fibrillation, DCM = dilated cardiomyopathy, HF = heart failure, NICM = non-ischemic cardiomyopathy, OR = odds ratio.

Fig S3. Enriched Reactome pathways and their corresponding -log 10(p-value) per cardiac outcome.

Figure S4. Heatmap displaying the percentage of overlapping associated proteins between metabolite pairs.

NB. Orange boxes represent clusters further studied in detail.

Abbreviations: a = acyl residue, aa = diacyl residue, ae = acyl-alkyl residue, LPC = lysophosphatidylcholine, PC = phosphatidylcholine, SM = sphingomyelin.

Fig S5. Annotated network of prioritised metabolites, proteins, and outcomes for which the metabolites have at least 20% associated common proteins belonging to the metabolite classes LPC and PC.

NB. Prioritised proteins are represented by circles, metabolites by diamonds, outcomes by triangles. Circle colours represent protein druggability, where drugged is defined as targeted by an approved compound and druggable as targeted by a compound, see Methods. Increasing effect is displayed by a red arrow, decreasing effect by a blue arrow. Abbreviations: a = acyl residue, aa = diacyl residue, AF = atrial fibrillation, HF = heart failure, LPC = lysophosphatidylcholine, PC = phosphatidylcholine.

Fig S6. Annotated network of prioritised metabolites, proteins, and outcomes for which the metabolites have at least 20% associated common proteins belonging to the metabolite class PC.

NB. Prioritised proteins are represented by circles, metabolites by diamonds, outcomes by triangles. Circle colours represent protein druggability, where drugged is defined as targeted by an approved compound and druggable as targeted by a compound, see Methods. Increasing effect is displayed by a red arrow, decreasing effect by a blue arrow. Abbreviations: ae = acyl-alkyl residue, AF = atrial fibrillation, HF = heart failure, PC = phosphatidylcholine.

Fig S7. Annotated network of prioritised metabolites, proteins, and outcomes for which the metabolites have at least 20% associated common proteins belonging to the metabolite classes LPC and PC.

NB. Prioritised proteins are represented by circles, metabolites by diamonds, outcomes by triangles. Circle colours represent protein druggability, where drugged is defined as targeted by an approved compound and druggable as targeted by a compound, see Methods. Increasing effect is displayed by a red arrow, decreasing effect by a blue arrow. Abbreviations: a = acyl residue, aa = diacyl residue, ae = acyl-alkyl residue, AF = atrial fibrillation, HF = heart failure, LPC = lysophosphatidylcholine, PC = phosphatidylcholine.

Fig S8. Enriched Reactome pathways and their corresponding -log 10(p-value) per metabolite cluster.

CAS = contact activation system (CAS); KKS = kallikrein/kinin system

Abbreviations: LPC = lysophosphatidylcholine, PC = phosphatidylcholine.

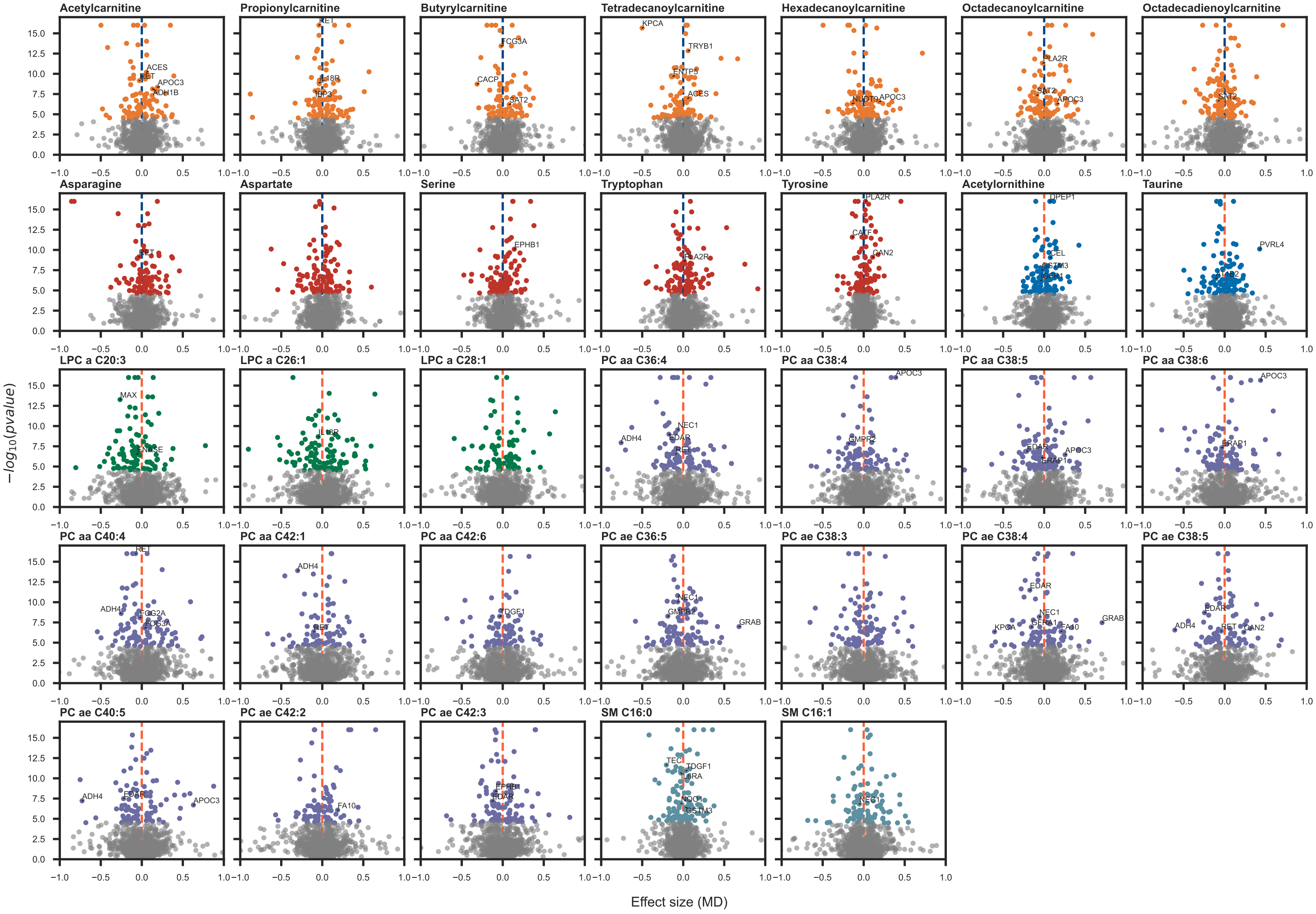
Acetylcarnitine
Propionylcarnitine
Butyrylcarnitine
Tetradecanoylcarnitine
Hexadecanoylcarnitine
Octadecanoylcarnitine
Octadecadienoylcarnitine
Asparagine
Aspartate
Serine
Tryptophan
Tyrosine
Acetylornithine
Taurine
LPC a C20:3
LPC a C26:1
LPC a C28:1
PC aa C36:4
PC aa C38:4
PC aa C38:5
PC aa C38:6
PC aa C40:4
PC aa C42:1
PC aa C42:6
PC ae C36:5
PC ae C38:3
PC ae C38:4
PC ae C38:5
PC ae C40:5
PC ae C42:2
PC ae C42:3
SM C16:0
SM C16:1
$-log_{10}(pvalue)$
Effect size (MD)

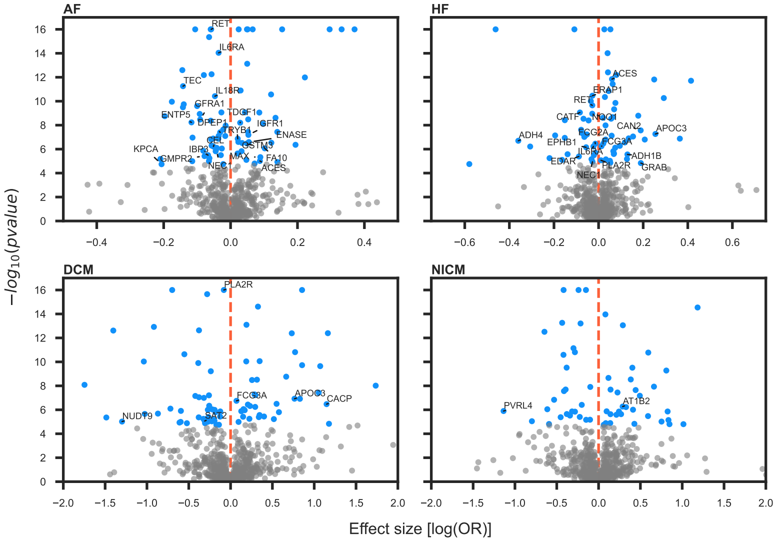
AF
HF
DCM
NICM
RET
IL6RA
TEC
IL18R
GFRA1
ENTP5
TDGF1
DPEP1
IGFR1
TRYB1
ENASE
CEL
GSTM3
KPCA
IBP3
MAX
FA10
GMPR2
NEC1
ACES
ERAP1
NQO1
CATF
CAN2
APOC3
ADH4
FCG2A
FCG3A
EPHB1
IL6RA
ADH1B
EDAR
PLA2R
GRAB
PLA2R
FCG3A
APOC3
CACP
NUDT9
SAT2
PVRL4
AT1B2
$-log_{10}(pvalue)$
Effect size [log(OR)]

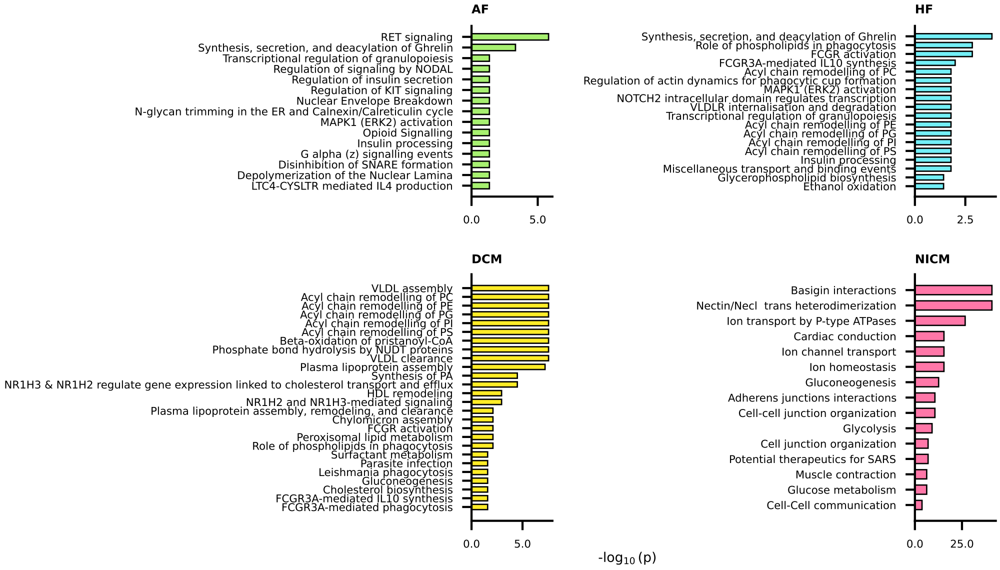
AF
RET signaling
Synthesis, secretion, and deacylation of Ghrelin
Transcriptional regulation of granulopoiesis
Regulation of signaling by NODAL
Regulation of insulin secretion
Regulation of KIT signaling
Nuclear Envelope Breakdown
N-glycan trimming in the ER and Calnexin/Calreticulin cycle
MAPK1 (ERK2) activation
Opioid Signalling
Insulin processing
G alpha (z) signalling events
Disinhibition of SNARE formation
Depolymerization of the Nuclear Lamina
LTC4-CYSLTR mediated IL4 production
0.0
5.0
HF
Synthesis, secretion, and deacylation of Ghrelin
Role of phospholipids in phagocytosis
FCGR activation
FCGR3A-mediated IL10 synthesis
Acyl chain remodelling of PC
Regulation of actin dynamics for phagocytic cup formation
MAPK1 (ERK2) activation
NOTCH2 intracellular domain regulates transcription
VLDLR internalisation and degradation
Transcriptional regulation of granulopoiesis
Acyl chain remodelling of PE
Acyl chain remodelling of PG
Acyl chain remodelling of PI
Acyl chain remodelling of PS
Insulin processing
Miscellaneous transport and binding events
Glycerophospholipid biosynthesis
Ethanol oxidation
0.0
2.5
DCM
VLDL assembly
Acyl chain remodelling of PC
Acyl chain remodelling of PE
Acyl chain remodelling of PG
Acyl chain remodelling of PI
Acyl chain remodelling of PS
Beta-oxidation of pristanoyl-CoA
Phosphate bond hydrolysis by NUDT proteins
VLDL clearance
Plasma lipoprotein assembly
Synthesis of PA
NR1H3 & NR1H2 regulate gene expression linked to cholesterol transport and efflux
HDL remodeling
NR1H2 and NR1H3-mediated signaling
Plasma lipoprotein assembly, remodeling, and clearance
Chylomicron assembly
FCGR activation
Peroxisomal lipid metabolism
Role of phospholipids in phagocytosis
Surfactant metabolism
Parasite infection
Leishmania phagocytosis
Gluconeogenesis
Cholesterol biosynthesis
FCGR3A-mediated IL10 synthesis
FCGR3A-mediated phagocytosis
0.0
5.0
NICM
Basigin interactions
Nectin/Necl trans heterodimerization
Ion transport by P-type ATPases
Cardiac conduction
Ion channel transport
Ion homeostasis
Gluconeogenesis
Adherens junctions interactions
Cell-cell junction organization
Glycolysis
Cell junction organization
Potential therapeutics for SARS
Muscle contraction
Glucose metabolism
Cell-Cell communication
0.0
25.0
-$\log_{10}$(p)

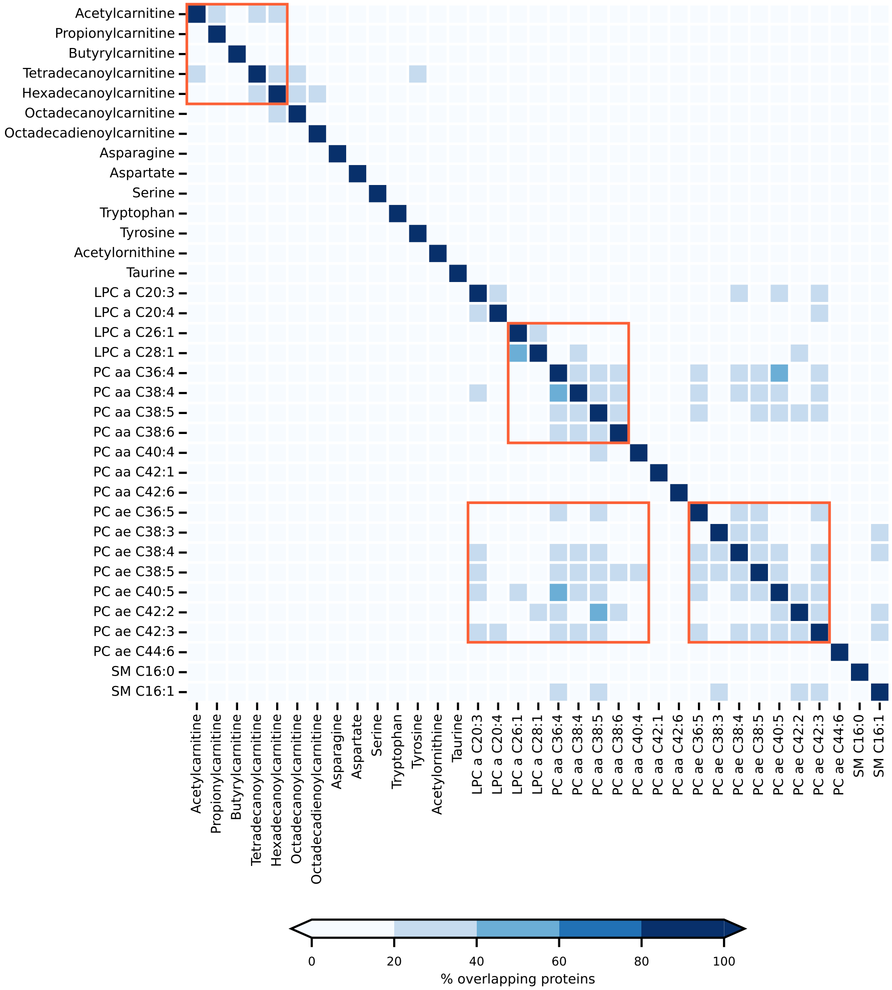
Acetylcarnitine
Propionylcarnitine
Butyrylcarnitine
Tetradecanoylcarnitine
Hexadecanoylcarnitine
Octadecanoylcarnitine
Octadecadienoylcarnitine
Asparagine
Aspartate
Serine
Tryptophan
Tyrosine
Acetylornithine
Taurine
LPC a C20:3
LPC a C20:4
LPC a C26:1
LPC a C28:1
PC aa C36:4
PC aa C38:4
PC aa C38:5
PC aa C38:6
PC aa C40:4
PC aa C42:1
PC aa C42:6
PC ae C36:5
PC ae C38:3
PC ae C38:4
PC ae C38:5
PC ae C40:5
PC ae C42:2
PC ae C42:3
PC ae C44:6
SM C16:0
SM C16:1
0
20
40
60
80
100
% overlapping proteins

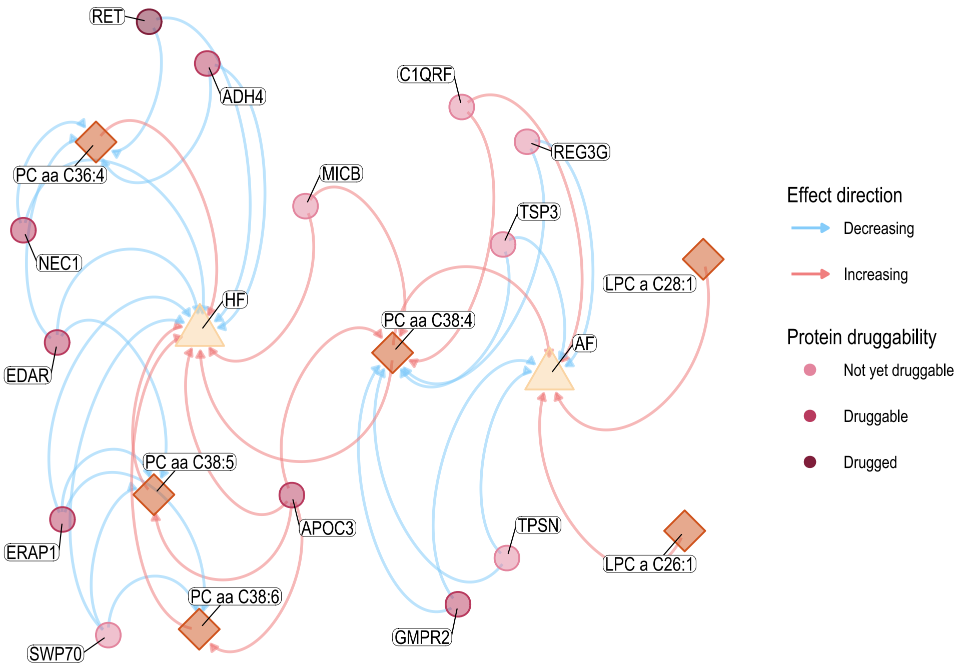
RET
ADH4
C1QRF
REG3G
MICB
TSP3
PC aa C36:4
NEC1
HF
PC aa C38:4
AF
LPC a C28:1
EDAR
PC aa C38:5
APOC3
ERAP1
TPSN
LPC a C26:1
PC aa C38:6
SWP70
GMPR2
Effect direction
Decreasing
Increasing
Protein druggability
Not yet druggable
Druggable
Drugged

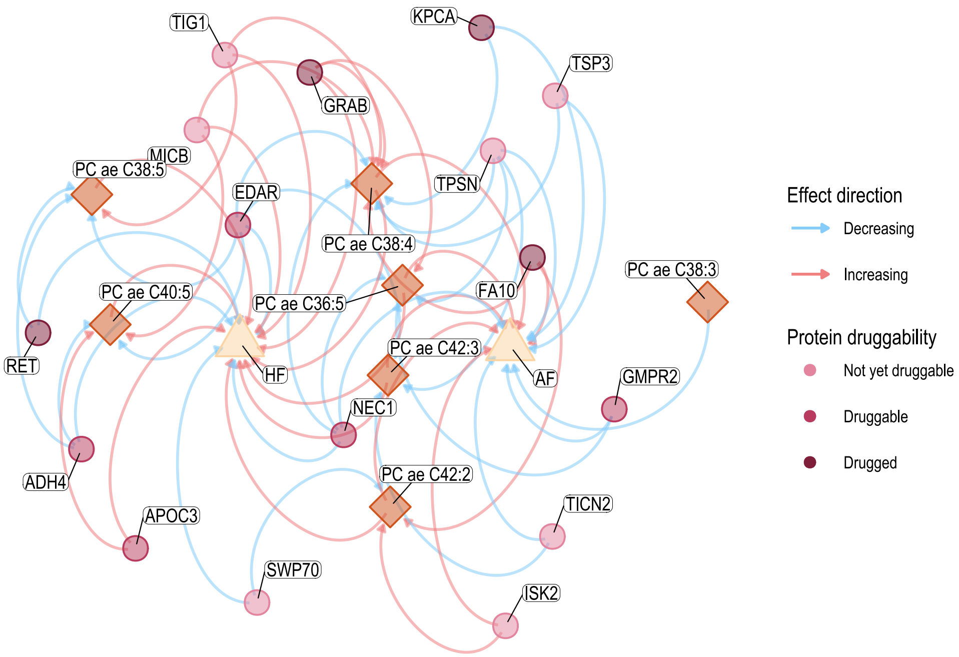
TIG1
KPCA
TSP3
GRAB
MICB
PC ae C38:5
EDAR
TPSN
PC ae C38:4
FA10
PC ae C38:3
PC ae C40:5
PC ae C36:5
PC ae C42:3
RET
HF
AF
GMPR2
NEC1
ADH4
PC ae C42:2
APOC3
TICN2
SWP70
ISK2
Effect direction
Decreasing
Increasing
Protein druggability
Not yet druggable
Druggable
Drugged

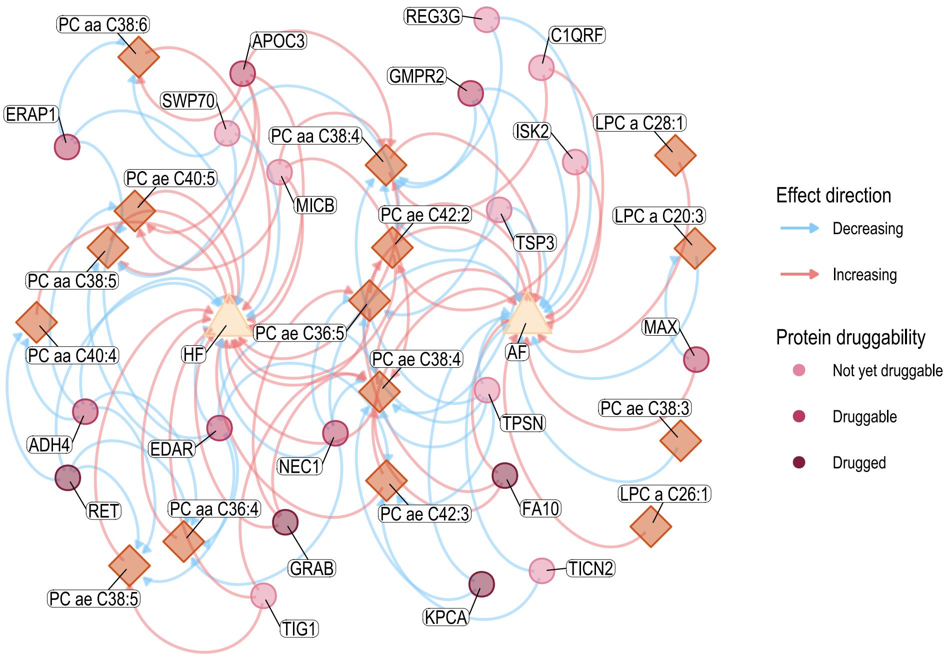
PC aa C38:6
APOC3
REG3G
C1QRF
GMPR2
SWP70
ERAP1
PC aa C38:4
ISK2
LPC a C28:1
MICB
PC ae C40:5
PC ae C42:2
TSP3
LPC a C20:3
PC aa C38:5
PC ae C36:5
MAX
PC aa C40:4
HF
AF
PC ae C38:4
TPSN
PC ae C38:3
ADH4
EDAR
NEC1
RET
PC aa C36:4
PC ae C42:3
FA10
LPC a C26:1
GRAB
TICN2
PC ae C38:5
KPCA
TIG1
Effect direction
Decreasing
Increasing
Protein druggability
Not yet druggable
Druggable
Drugged

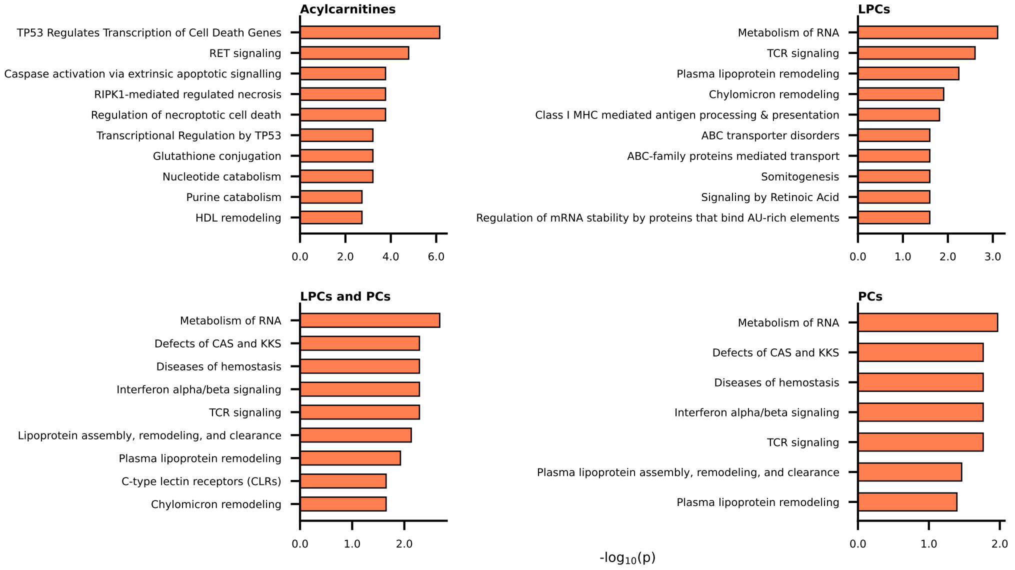

Acylcarnitines
TP53 Regulates Transcription of Cell Death Genes
RET signaling
Caspase activation via extrinsic apoptotic signalling
RIPK1-mediated regulated necrosis
Regulation of necroptotic cell death
Transcriptional Regulation by TP53
Glutathione conjugation
Nucleotide catabolism
Purine catabolism
HDL remodeling
0.0
2.0
4.0
6.0
LPCs
Metabolism of RNA
TCR signaling
Plasma lipoprotein remodeling
Chylomicron remodeling
Class I MHC mediated antigen processing & presentation
ABC transporter disorders
ABC-family proteins mediated transport
Somitogenesis
Signaling by Retinoic Acid
Regulation of mRNA stability by proteins that bind AU-rich elements
0.0
1.0
2.0
3.0
LPCs and PCs
Metabolism of RNA
Defects of CAS and KKS
Diseases of hemostasis
Interferon alpha/beta signaling
TCR signaling
Lipoprotein assembly, remodeling, and clearance
Plasma lipoprotein remodeling
C-type lectin receptors (CLRs)
Chylomicron remodeling
0.0
1.0
2.0
PCs
Metabolism of RNA
Defects of CAS and KKS
Diseases of hemostasis
Interferon alpha/beta signaling
TCR signaling
Plasma lipoprotein assembly, remodeling, and clearance
Plasma lipoprotein remodeling
0.0
1.0
2.0
$-\log_{10}(p)$
